# Ascorbic acid enhances *in vitro* primordial germ cell-like cell differentiation from mouse ESCs

**DOI:** 10.3389/fcell.2026.1755998

**Published:** 2026-03-04

**Authors:** Umesh Kumar, Nithyapriya Kumar, P. Chandra Shekar, Kumarasamy Thangaraj

**Affiliations:** CSIR-Centre for Cellular and Molecular Biology (CCMB), Hyderabad, India

**Keywords:** BMP signaling, DPPA3 (Stella), epigenetic reprogramming, germ cell specification, *in vitro* germ cell differentiation, mESCs, PGCLCs, TET-mediated DNA demethylation

## Abstract

**Introduction:**

Primordial germ cells (PGCs), the precursors of sperm and oocytes, are specified from a subset of epiblast cells during the post-implantation stage of mammalian embryonic development. Over the past decade, primordial germ cell-like cells (PGCLCs) have been successfully generated *in vitro* from mouse and human embryonic stem cells (ESCs). *In vitro* PGCLC differentiation provides a powerful system to study germ cell specification and epigenetic reprograming. Notably, mouse PGCLCs can further mature into functional sperm following transplantation into neonatal testes. Despite these advancements, in vitro PGCLC differentiation remains inefficient, highlighting gaps in our understanding of PGC specification.

**Methods:**

To identify strategies for improving differentiation efficiency, we generated a DPPA3-mCherry PGC reporter mouse ESC line (TDM11) using CRISPR–Cas9-mediated knock-in. We implemented an embryoid body (EB)-based differentiation strategy under a non-adherent, defined culture condition, which systematically examined factors influencing PGCLC specification. PGCLC specification was assessed temporarily by flow cytometer-based quantification of DPPA3-mCherry expressing cells. Given the established role of ten–eleven translocation 1 (TET1)-mediated epigenetic regulation in PGC development, we evaluated that ascorbic acid (AA), a known activator of TET1, acts as a potent enhancer of PGCLC induction from ESCs.

**Results:**

Flow cytometric analysis revealed a substantial enrichment of DPPA3-mCherry-positive PGCLC upon AA and transferrin supplementation compared to standard EB medium. The PGCLC specification was further enhanced by combined supplementation with BMP4 and BMP8B in AA- and transferrin-supplemented basal EB medium. The DPPA3-mCherry PGCLC further characterize for the expression of other PGC-specific genes and successful derivation of embryonic germ cells.

**Discussion:**

Building upon this finding, we established a highly efficient and reproducible protocol for in vitro PGCLC differentiation from mouse embryonic stem cells (mESCs) by modulating epigenetic regulation through AA. This system provides a valuable platform for dissecting the molecular mechanism and epigenetic reprograming during early germ cell development and potential therapeutic applications.

## Introduction

Infertility is a major and growing health concern, affecting approximately one in six couples over their lifetime (WHO, 2025). Notably, male-related factors contribute to nearly 50% of infertility cases. Male infertility most commonly arises from semen ejaculation dysfunction, abnormal sperm morphology, or low or complete absence of sperm in the ejaculate (azoospermia or severe oligozoospermia) (Barone et al., 2026). These conditions may be caused by genetic variants, environmental factors, or a combination of both (Kumar et al., 2024; Sudhakar et al., 2023; Sudhakar et al., 2019; Segal and Giudice, 2019). Current assisted reproductive technologies (ARTs), including *in vitro* fertilization (IVF) and intracytoplasmic sperm injection (ICSI), fundamentally rely on the presence of functional sperm (Barone et al., 2026). As a result, these approaches are ineffective for a substantial subset of infertile men who completely lack sperm in the ejaculate, leaving them without options for biological parenthood. This clinical limitation has intensified interest in alternative strategies, such as *in vitro* gametogenesis, aimed at generating functional germ cells *ex vivo* (Esfahani et al., 2024).

Germ cells are the precursors of gametes, which are responsible for genome recombination and genetic transmission to the next generation (Kurimoto et al., 2008). In mammals, germ cell development begins with unipotent primordial germ cells (PGCs), which undergo sequential differentiation to form sperm in male subjects and ova in female subjects (Yu et al., 2025). Fertilization of these gametes results in a totipotent zygote, whose totipotency gradually diminishes during the transition from blastomere to blastocyst. In mice and humans (and presumably all mammals), germ cell lineage specification occurs in the early embryo through epigenesis, where a subset of epiblast cells is induced by paracrine signaling from surrounding tissues (de Sousa Lopes et al., 2007; Kurimoto et al., 2008). Although key transcriptional regulators of PGC specification have been identified, the extrinsic signals and regulatory mechanisms that ensure efficient germ cell induction remain incompletely defined.

Direct investigation of human germ cell development is severely constrained by limited access to early embryonic material. Stem cell-based *in vitro* gametogenesis has therefore emerged as a powerful model for studying germ cell fate decisions. Landmark studies demonstrated the derivation of PGC-like cells (PGCLCs) and functional gametes from embryonic stem cells (ESCs) and induced pluripotent stem cells in mice and humans (Hayashi et al., 2011; Hikabe et al., 2016; Yamashiro et al., 2020). These breakthroughs offer potential applications in regenerative medicine to restore fertility (Sosa et al., 2018). Despite these advances, current PGCLC differentiation protocols remain complex, inefficient, and heterogeneous, limiting mechanistic studies and translational potential.

Vitamin C (ascorbic acid, AA) acts as a cofactor for ten–eleven translocation 1 (TET1), a DNA dioxygenase that plays a key role in epigenetic reprogramming during PGC development. Consistent with this, AA has been shown to enhance stem cell differentiation through its effects on chromatin remodeling and DNA demethylation. Li et al. (2019) reported that AA promotes early germ cell specification from human ESCs. However, these studies did not establish a simplified or high-efficiency for differentiating PGCLCs from mouse ESCs. Moreover, the combinatorial effects of AA with defined BMP signaling cues during early PGC induction in mouse remain poorly characterized.

We aimed to establish an efficient and simplified protocol for differentiating PGCLCs from mouse ESCs. To facilitate PGCLC identification, we generated a DPPA3-mCherry reporter mouse ESC line using CRISPR–Cas9-mediated knock-in (Ohinata et al., 2008). Using this system, we optimized a minimal differentiation medium, containing bovine serum, AA, transferrin, BMP4, and BMP8b. Under these conditions, approximately 48% cells acquired DPPA3-mCherry-positive PGCLCs identity by day 5 of differentiation. Our approach provides a robust, reporter-based platform that improves efficiency and simplicity over existing methods, facilitating mechanistic studies on germ cell development and advancing *in vitro* gametogenesis toward translational applications.

## Results

### Generation of DPPA3 reporter mESC through CRISPR–Cas9-mediated knock-in

**FIGURE 1:**
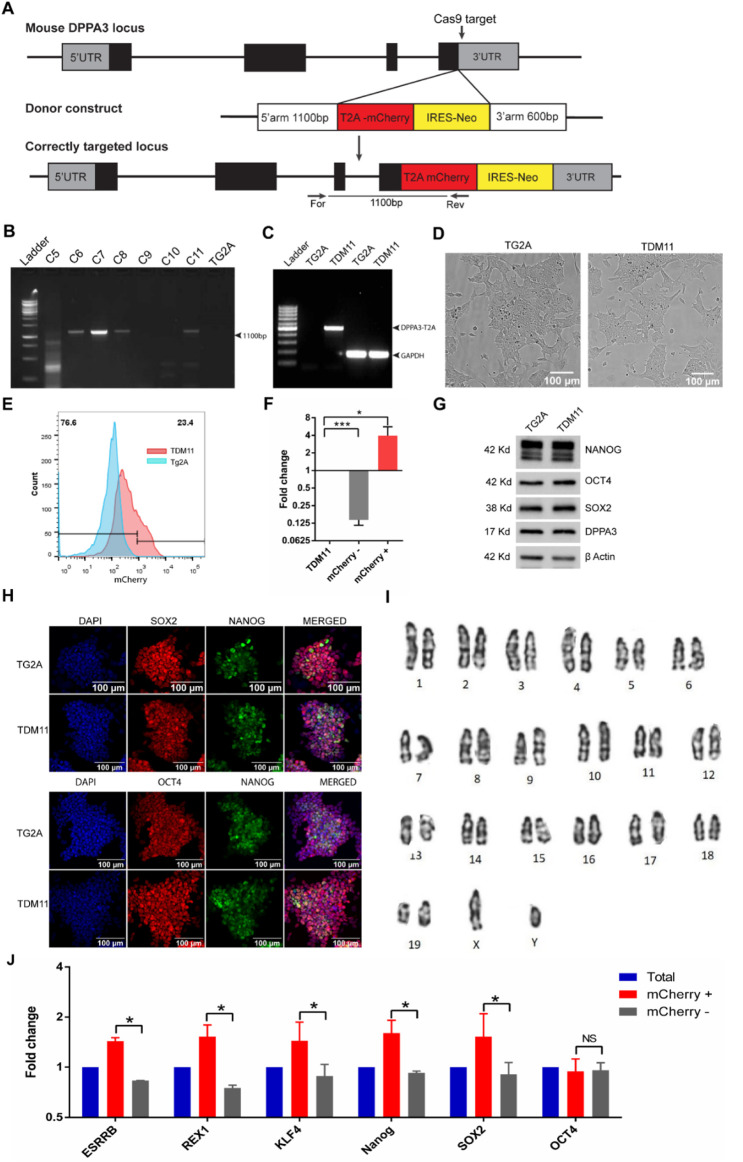
Creation and characterization of TDM11 cell line. **(A)** Schematic representation of the targeting vector specific for the last exon of *Dppa3* and the knock-in strategy. The primers (For, genome-specific forward; Res, targeting vector-specific reverse) used for PCR-based screening of correctly integrated clones are indicated. **(B)** PCR-based screening of neomycin-resistant clones for homologous recombination using a forward primer from the *Dppa3* locus (outside the homologous arm) and a reverse primer from the T2A-mCherry sequence. Clone number 11 was identified as positive and designated TDM11. **(C)** Amplification of the *DPPA3*-T2A-mCherry transcript from cDNA of clone 11 (TDM11) using a forward primer from the first exon of *Dppa3* (untargeted sequence) and a reverse primer from the T2A sequence. **(D)** Microscopic analysis of colony morphology of TDM11 cells compared with the parental TG2A cells. **(E)** Flow cytometric analysis of mCherry fluorescence in TDM11 cells cultured in LIF + serum compared with TG2A-negative control cells. **(F)** qPCR analysis of *Dppa3* expression in mCherry-positive and -negative sorted cells compared to the total TDM11 population. **(G)** Western blot and **(H)** immunofluorescence analysis of pluripotency markers OCT4, SOX2, and NANOG in TDM11 compared with TG2A cells. Uncropped blots are provided in the Supplementary Figure S3. **(I)** Karyotype analysis of the TDM11 cell line. **(J)** qPCR analysis of naïve pluripotency markers in mCherry-positive and -negative cells compared to the total TDM11 cell population.

We generated a DPPA3-mCherry reporter mouse embryonic stem cell (mESC) line, named TDM11, using CRISPR–Cas9-mediated knock-in, as described in the *Materials and Methods* section (Figures 1A–C; Supplementary Figure S1). Microscopic analysis revealed that TDM11 cells were morphologically identical to the parental TG2A cell line (Figure 1D). In the presence of the leukemia inhibitory factor (LIF) and serum, approximately 23% of TDM11 cells exhibited mCherry fluorescence (Figure 1E), corroborating previous studies (Hayashi et al., 2008).

Fluorescence-activated cell sorting (FACS) of mCherry-positive cells showed a substantial enrichment of Dppa3 expression (4-fold), whereas mCherry-negative cells exhibited a marked decrease (0.14-fold) compared to the unsorted total TDM11 cells (Figure 1F). These results confirm that mCherry fluorescence reliably reports the DPPA3 expression.

Furthermore, the expression levels and profiles of pluripotency markers OCT4, SOX2, and NANOG in TDM11 cells were identical to those in the parental TG2A cell line (Figures 1G,H), confirming the pluripotent nature of TDM11. Additionally, the expression of DPPA3 in TDM11 cells was comparable to that in TG2A cells (Figure 1G), indicating that the T2A-mCherry-IRES-Neo knock-in at the *Dppa3* locus does not disrupt endogenous DPPA3 protein expression.

Karyotype analysis after 10 passages confirmed that TDM11 cells remained euploid (Figure 1I), suggesting genomic stability.

### DPPA3-mCherry-positive ESCs are poised toward naïve state of pluripotency

DPPA3 expression was heterogeneous in ESCs, with approximately 23% of cells expressing DPPA3-mCherry. To assess the molecular identity of DPPA3-positive cells in undifferentiated ESCs, we sorted DPPA3-mCherry-positive and -negative cell populations and performed qPCR for naïve state ESC markers and pluripotency factors. The results showed that DPPA3-mCherry-positive cells expressed significantly higher levels of naïve-stage markers, including Klf4, Rex1, and Esrrb, as well as the pluripotency factors Sox2 and Nanog, than DPPA3-mCherry-negative cells (Figure 1J). Moreover, Oct4 expression was similar in both populations (Figure 1J). These results clearly show that the DPPA3-mCherry-positive ESC population is poised toward the naïve pluripotency state, consistent with previous studies (Hayashi et al., 2008).

### *In vitro* differentiation of ESCs into PGC-like cells

We differentiated TDM11 cells under embryoid body (EB) culture conditions using basal medium (Table 1) and analyzed the percentage of DPPA3-mCherry-positive cells during differentiation by flow cytometry (Supplementary Figure S2A). Our results showed that DPPA3 expression decreased during the first 2 days of differentiation, with a subsequent increase beginning on day 3 (Supplementary Figure S2B). By day 5, approximately 26% of the cells were DPPA3-mCherry-positive, suggesting that they had acquired PGCLC characteristics (Supplementary Figure S2B).

**TABLE 1:** Medium composition screened for PGCLC differentiation.

Basal medium	Basal medium plus ascorbic acid	Differentiation medium	Cytokine-supplemented differentiation medium
GMEM, 10% fetal bovine serum, 0.1 mM nonessential amino acid, 0.1 mM mercaptoethanol, 1 mM sodium pyruvate, and 1 mM GlutaMAX	GMEM, 10% fetal bovine serum, 0.1 mM nonessential amino acid, 0.1 mM mercaptoethanol, 1 mM sodium pyruvate, and 1 mM GlutaMAX +50 μg/mL ascorbic acid	GMEM, 10% fetal bovine serum, 0.1 mM nonessential amino acid, 0.1 mM mercaptoethanol, 1 mM sodium pyruvate, and 1 mM GlutaMAX +50 μg/mL ascorbic acid +200 μg/mL transferrin (partially iron-saturated cat #T8158 sigma)	GMEM, 10% fetal bovine serum, 0.1 mM nonessential amino acid, 0.1 mM mercaptoethanol, 1 mM sodium pyruvate, and 1 mM GlutaMAX +50 μg/mL ascorbic acid +200 μg/mL transferrin +100 ng/mL BMP4 + 50 ng/mL BMP8b or GMEM, 10% fetal bovine serum, 0.1 mM nonessential amino acid, 0.1 mM mercaptoethanol, 1 mM sodium pyruvate, and 1 mM GlutaMAX +50 μg/mL ascorbic acid +200 μg/mL transferrin +1000 unit/mL LIF

These findings indicate that PGCLC specification can occur alongside differentiation into the three germ lineages within EB culture conditions. Furthermore, mESCs cultured under LIF plus serum conditions retain the competence to differentiate into PGCLCs *in vitro*.

### Ascorbic acid and transferrin supplementation enhances PGCLC differentiation

Given the reported role of AA in supporting TET1-mediated epigenetic reprograming in PGC, we investigated the effect of AA supplementation in basal medium (Table 1) on PGCLC differentiation efficiency. Using our model system, we analyzed the abundance of DPPA3-mCherry-positive cells daily for up to 8 days using flow cytometry (Supplementary Figure S2B). Interestingly, AA supplementation markedly increased the proportion of DPPA3-mCherry-positive cells during differentiation (Supplementary Figure S2B). As expected, during the first 2 days, the abundance of DPPA3-mCherry-positive cells decreased compared to that of undifferentiated ESCs. However, beginning on day 3, we observed a strong induction of DPPA3-mCherry expression within EBs (Supplementary Figure S2B). By day 5, approximately 34% of the cells under AA-supplemented conditions were DPPA3-mCherry-positive, indicating enhanced PGCLC specification (Supplementary Figure S2B). Beyond day 5, EBs continued to grow, but the proportion of DPPA3-mCherry-positive cells gradually decreased each day until day 8.

Furthermore, we examined the effect of transferrin supplementation on PGCLC specification. Notably, the addition of partial iron-saturated transferrin to AA-supplemented basal medium, hereafter referred to as the differentiation medium (Table 1), further enhanced differentiation efficiency. By day 5, approximately 40% of the cells in EBs were DPPA3-mCherry-positive (Supplementary Figure S2B), demonstrating an improved induction of PGCLCs. After day 5, DPPA3-mCherry expression gradually decreased, following a pattern similar to that observed in EBs cultured in basal medium and AA-supplemented medium.

### BMP signaling cascade in PGCLC specifications

**FIGURE 2:**
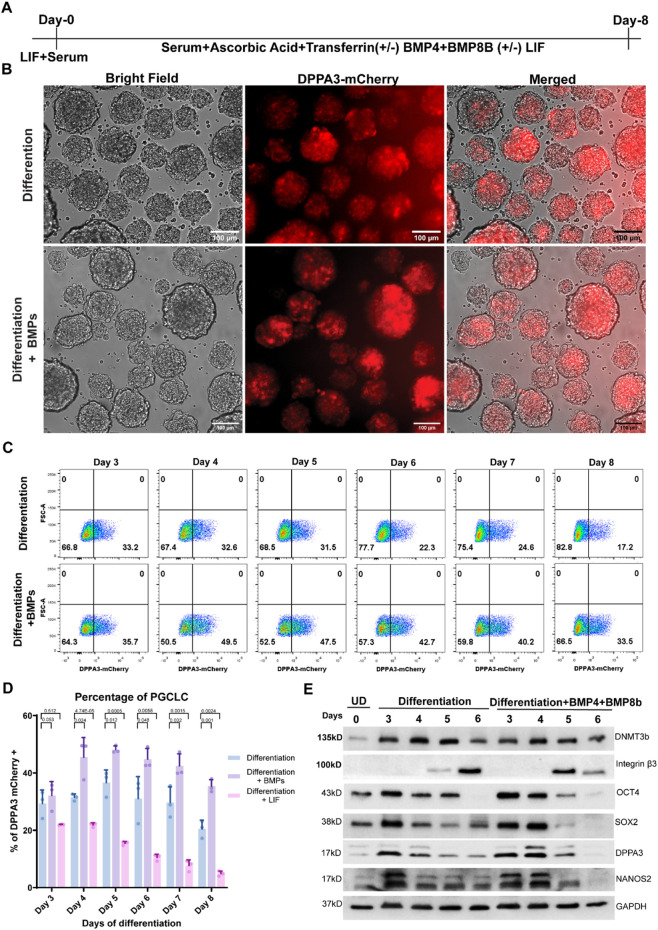
PGCLC induction from TDM11 mESCs and characterization. **(A)** Schematic overview of the strategy used for continuous PGCLC induction from day 0 to day 8 from TDM11 mouse embryonic stem cells (mESCs), with indicated factors (ascorbic acid, transferrin, BMP4, BMP8b, and LIF) maintained throughout differentiation. **(B)** Representative fluorescence (DPPA3-mCherry) and brightfield images of embryoid bodies (EBs) at day 4 of differentiation cultured in differentiation medium with or without BMP4 and BMP8b supplementation. **(C)** Flow cytometric analysis of DPPA3-mCherry-positive cells from day 3 to day 8 of differentiation. **(D)** Quantification of DPPA3-mCherry-positive cells in EBs differentiated in cytokine-supplemented medium compared with differentiation medium alone (day 4 to day 7). Data represent three independent biological replicates per condition. Each data point represents an individual sample. Statistical significance was assessed using a *t*-test, and *P* < 0.05 was considered statistically significant; *P*-values are indicated above the bars. **(E)** Western blot analysis of PGC marker expression during differentiation, in EBs differentiated in differentiation medium or in differentiation + BMP4+BMP8 (BMPs) medium compared with undifferentiated (UD) TDM11 cells (day 0). Uncropped blots are provided in the Supplementary Figure S4.

To examine whether the PGCLCs differentiated in our defined system (in differentiation medium) are responsive to these cytokines and if we can enhance the efficiency of PGCLC specification further, we supplemented the differentiation medium with the well-defined recombinant growth factors, BMP4 and BMP8b (described in Table 1; Figure 2A) (Hayashi et al., 2011; Ying et al., 2001). Indeed, we found a significant increase in DPPA3-mCherry-positive cells in EBs after the supplementation of BMP4 and BMP8b in the differentiation medium from day 3 to day 8 (Figures 2B,C). For example, on the fifth day of differentiation, approximately 48% cells were DPPA3-mCherry-positive in EBs differentiated into the BMP4- and BMP8B-supplemented differentiation medium, which is markedly high compared to EBs grown in the differentiation medium alone (36%) (Figures 2C,D).

**FIGURE 3:**
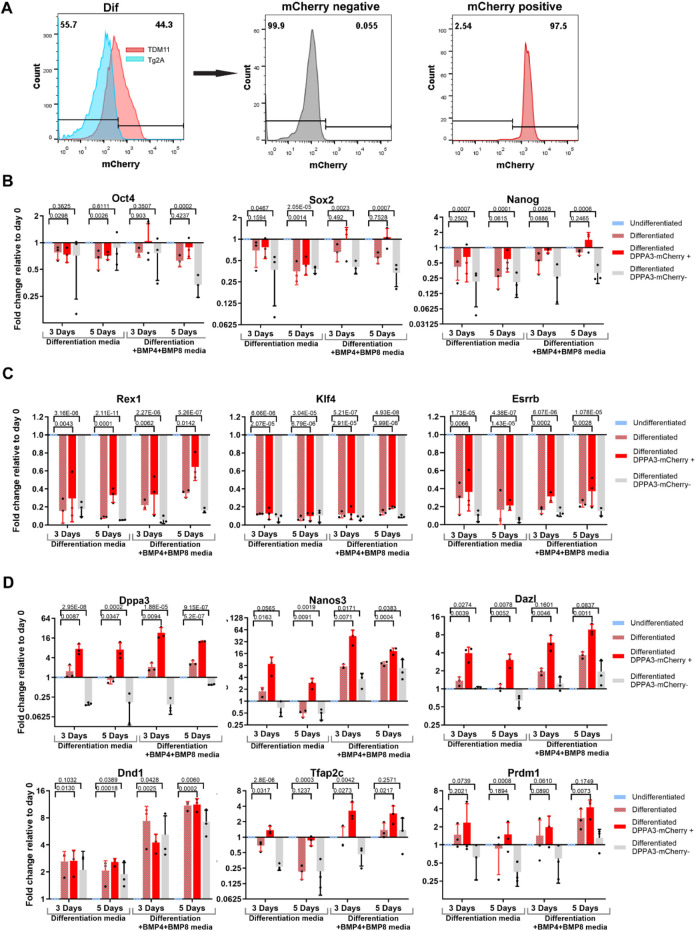
Molecular identity of *in vitro* differentiated PGCLCs. **(A)** Representative flow cytometry analysis showing sorting of DPPA3-mCherry-positive and -negative cells from differentiating embryoid bodies (EBs). **(B)** qPCR analysis of PGC-associated pluripotency factors in DPPA3-mCherry-positive and -negative sorted cells from EBs differentiated in differentiation medium or in differentiation + BMP4+BMP8 medium at days 3 and 5 compared with undifferentiated ESCs (day 0). **(C)** qPCR analysis of naïve pluripotency factors in DPPA3-mCherry-positive and -negative sorted cells from EBs differentiated in differentiation medium or in differentiation + BMP4+BMP8 medium at days 3 and 5 compared with undifferentiated ESCs. **(D)** qPCR analysis of PGC-specific genes in DPPA3-mCherry-positive and -negative sorted cells from EBs differentiated in differentiation medium or in differentiation + BMP4+BMP8 medium at days 3 and 5 compared with undifferentiated ESCs. Data in **(B–D)** represent three independent biological replicates per condition. Each data point represents an individual sample. Statistical significance **(B,C)** was assessed using a two-tailed Student’s t-test, whereas in **(D)**, using a one-tailed Student’s test; *P* < 0.05 was considered statistically significant, and *P*-values are indicated above the bars.

Overall, DPPA3-mCherry-positive cells began to increase by day 3, peaked at day 5, and then gradually decreased thereafter in every medium composition (Supplementary Figure S2; Figures 2C,D). By day 8, ∼17% of cells were DPPA3-mCherry-positive in the differentiation medium alone, whereas BMP-supplemented EBs contained ∼33% DPPA3-mCherry-positive cells (Figures 3C,D). These results demonstrate that PGCLC differentiation in our system is responsive to PGC-specific BMP signaling.

These results were further confirmed by Western blotting of DPPA3 at every day after the third day of differentiation (Figure 2E). Compared to that in undifferentiated cells, the DPPA3 expression markedly increased on the third day of differentiation and then gradually decreased in EBs differentiated in only the differentiation medium, whereas the DPPA3 expression continued increasing until the fourth day of differentiation, where BMP4 and BMP8b were supplemented in the differentiation medium (Figure 2E). Moreover, we also examined the expression profile of other germ cell markers, including NANOS2, OCT4, SOX2, and Integrin β3. As expected, the expression profile of the PGCLC marker NANOS2 was similar to that of DPPA3, whereas the migratory PGC marker Integrin β3 began to be expressed on the fifth day of differentiation. Integrin β3 expression in EBs differentiated in BMP4- and BMP8b-supplemented differentiation medium was markedly higher than in EBs differentiated in differentiation medium alone on the fifth day of differentiation (Figure 2E).

### Effects of LIF in PGCLC specifications

To investigate the role of LIF in the specification of PGCLCs within our differentiation system, as used in previously described protocols (Hayashi et al., 2011), we added LIF to the differentiation medium, as detailed in Table 1. We observed that the percentage of DPPA3-mCherry-positive cells decreased steadily from day 3 to day 8 of differentiation. By the fifth day, only approximately 15% of the cells were DPPA3-mCherry-positive, which is roughly three times lower than EBs cultured without LIF supplementation in the differentiation medium (Figure 2D). LIF is known to maintain ESCs in an undifferentiated state. These findings suggest that LIF inhibits PGC specification by preventing ESCs from differentiating in our protocol.

### Molecular characterization of derived PGCLC

To examine the molecular characteristics of DPPA3-mCherry-positive cells within the EBs, we used FACS to sort the DPPA3-mCherry-positive and -negative cells from differentiated EBs on day 3 or 5 of differentiation, cultured either in differentiation medium or in differentiation medium supplemented with BMP4 and BMP8b (Figure 3A). We analyzed the expression of PGC marker genes in DPPA3-mCherry-positive and -negative cells compared to undifferentiated TDM11 cells. The pluripotency factors Oct4, Sox2, and Nanog which also serve as PGC markers, were expressed equally in DPPA3-mCherry-positive PGCLCs (in particular, which derived from EBs differentiated in BMP4- and BMP8b-supplemented differentiation medium) and undifferentiated ESCs (Figure 3B). As expected, these markers were markedly downregulated in the DPPA3-mCherry-negative differentiated cells compared to those in undifferentiated cells (Figure 3B). However, Oct4 remained equally expressed in both positive and negative cells at days 3 and 5 in the differentiation medium and only at day 3 in the BMP4- and BMP8b-supplemented differentiation medium (Figure 3B), possibly due to its expression in other unexamined cell lineages.

As DPPA3 is also expressed in undifferentiated ESCs at the naïve stage (as shown in Figure 1H), we evaluated naïve-state ESC markers Klf4, Rex1, and Esrrb to ensure that the DPPA3-mCherry-positive cells in differentiating EBs do not belong to naïve-stage ES cells. All three markers were markedly downregulated (expressing almost null) in both DPPA3-mCherry-positive and -negative cells compared to those in undifferentiated ESCs, as presented in Figure 3C. These results show that DPPA3+ cells in EBs do not belong to the naïve state of ESCs.

We further assessed the expression of PGC-specific genes such as Dppa3, Dazl, and Nanos3, which were markedly enriched in DPPA3-mCherry-positive cells compared to undifferentiated cells (Figure 3D), whereas DPPA3-mCherry-negative cells either showed markedly downregulated expression compared to undifferentiated cells or low expression compared to DPPA3-mCherry-positive cells (Figure 3D). Dnd1, another PGC-expressing gene, showed marked upregulation in DPPA3-mCherry-positive cells compared to those in undifferentiated cells although it was also equally expressed in DPPA3-mCherry-negative cells (Figure 3D). The expression levels of Prdm1 and Tcfap2C in DPPA3-mCherry-positive cells were markedly upregulated (in particular, which derived from BMP4- and BMP8b-supplemented medium) compared to those in undifferentiated cells (Figure 3D), reinforcing our conclusion that Dppa3-mCherry-positive cells in differentiating EBs are indeed PGCLCs.

### Functional characterization of PGCLCs

**FIGURE 4:**
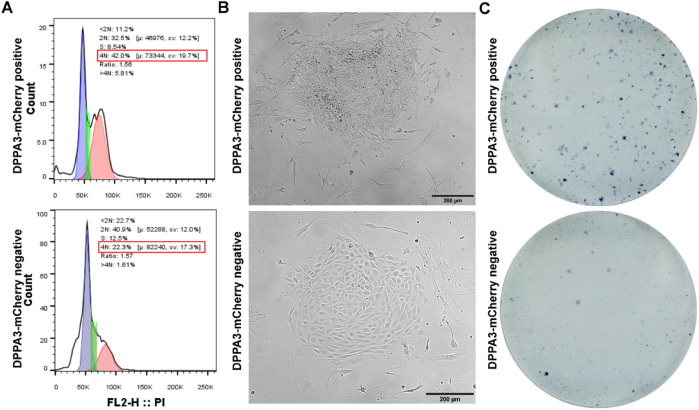
Functional characterization of DPPA3-mChery-positive PGCLC. **(A)** Cell cycle analysis of *DPPA3*-mCherry-positive and -negative PGCLCs sorted from day 4 EBs differentiated in differentiation medium. **(B)** Morphology of colonies derived from *DPPA3*-mCherry-positive and -negative PGCLCs cultured in EGC medium. **(C)** Alkaline phosphatase staining of colonies generated from *DPPA3*-mCherry-positive and -negative PGCLCs in EGC medium.

Cell cycle analysis revealed that Dppa3-mCherry-positive cells, sorted through FACS on the fifth day of differentiation, were enriched in the G2 phase (42%) compared to the negative cells at the same stage (22%) (Figure 4A). This suggests that DPPA3-mCherry-positive PGCLCs proliferate more slowly after induction in EBs, whereas negative cells continue to proliferate. This also explains the reduced abundance of Dppa3-mCherry-positive cells after the fifth day of differentiation (Figure 2C).

For further functional assessment, we cultured the sorted Dppa3-mCherry-positive and -negative cells from EBs, differentiated in the differentiation medium at day 5 on mouse embryonic fibroblasts (MEFs) using the embryonic germ cell (EGC) medium, as described by Vincent et al. (2011). In the presence of retinoic acid (RA, a component of EGC medium), PGCs form alkaline phosphatase-positive colonies, whereas undifferentiated ESCs differentiate into various somatic lineages (Dyce et al., 2018; Zhang et al., 2015). DPPA3-mCherry-positive sorted PGCLCs from the fifth day of differentiation formed stem cell-like colonies, whereas the negative cells differentiated into flat cuboidal cells (Figure 4B). Moreover, colonies derived from positive PGCLCs exhibited strong alkaline phosphatase staining, contrary to the unstained colonies derived from negative cells (Figure 4C). We also established continuously growing EGC cell lines from colonies developed from positive PGCLCs in the EGC medium. These findings confirm that the derived PGCLCs functionally resemble *in vivo* PGCs.

## Discussion

PGC specification in mammals, including mice and humans, involves three key processes: suppression of somatic genes, regaining potential pluripotency and latent totipotency, and extensive epigenetic reprogramming. These processes are driven by cytokine signaling. Recent studies have successfully recreated PGC pathways *in vitro* from pluripotent stem cells, using temporal manipulation of signaling pathways through cytokines and growth factors (Hayashi et al., 2011; Hikabe et al., 2016; Nakaki et al., 2013). When *in vitro*-derived PGCLCs are transplanted into neonatal testes, they can differentiate into functional gametes (Sosa et al., 2018).

In this study, a novel DPPA3-mCherry reporter mouse ESC line was created by inserting a T2A-mCherry-IRES-Neo cassette into the DPPA3 gene locus (Ohinata et al., 2008). This TDM11 cell line accurately reflects DPPA3 gene expression. Approximately 23% of ESCs in the LIF plus serum medium express the DPPA3 gene, consistent with previous studies (Hayashi et al., 2008).

EBs, which are 3D structures formed by aggregating ESCs, can mimic early embryonic development and contain PGCs, along with other cell lineages. In our experiments, approximately 25% of cells in 5-day EBs were DPPA3-mCherry-positive. During PGC development, extensive epigenetic reprogramming occurs, facilitated by enzymes such as the TET family, which are involved in DNA demethylation (Guo et al., 2017; Miyoshi et al., 2016; Seisenberger et al., 2012; Sekl et al., 2007; Wu and Zhang, 2011; Yamaguchi et al., 2013). The Prdm14, an early PGC expression factor, promotes DNA demethylation through TET enzyme activation (Okashita et al., 2016). AA acts as a cofactor for TET enzymes, enhancing germ cell development (DiTroia et al., 2019). Recently, it was demonstrated that maternal AA plays an important role in germ cell development and that maternal deficiency leads to a reduced number of germ cells (DiTroia et al., 2019). Moreover, AA directly activates TET enzymes in ESCs and leads to activate the expression of the germ cell-specific genes such as *Dazl* (Blaschke et al., 2013). Similarly, transferrin, secreted by sertoli cells, supports PGC self-renewal, as indicated in chickens and possibly applicable in mice (Gelly et al., 1994; Whyte et al., 2015; Conley et al., 2004).

We examined the effects of ascorbic acid and transferrin on EB-mediated PGC differentiation by monitoring DPPA3-mCherry expression. Ascorbic acid markedly increased the number of DPPA3-mCherry-positive cells (Supplementary Figure S2). Although our results are consistent with previous reports suggesting that AA activates TET1 and enhances PGC differentiation through TET1-mediated epigenetic reprogramming, we did not directly assess TET1 activity or perform loss-of-function experiments; therefore, this mechanistic link remains to be experimentally validated. BMP4 and BMP8b, critical for *in vivo* PGC specification, further enhanced the abundance of these cells when added in the differentiation medium (Figure 2D). The similar sizes of EBs across conditions suggest that the observed increase in PGCLC percentage likely reflects a combination of enhanced PGCLC differentiation and general effects on cell survival, rather than solely a broad effect on proliferation alone. DPPA3-mCherry-positive cells in ascorbic acid-supplemented EBs (both in differentiation medium and in that supplemented with BMPs) expressed important PGC markers such as Nanos3, DazL, and Dnd1 and retained pluripotency factors such as Oct4, Nanog, and Sox2 (Figures 3B,D). The inner cell mass (ICM) markers Rex1, Klf4, and Esrrb were markedly downregulated in DPPA3-mCherry-positive PGCLCs compared with undifferentiated ESCs, while the expression of Prdm14 and Tfap2C, common factors shared between ESCs and PGCs (Figures 3C,D), was retained, confirming the germ cell identity of PGCLCs. We observed that integrin β3, a characteristic marker of migrating PGCs, was induced earlier in BMP-supplemented differentiation medium than in the differentiation medium alone (Figure 2E).

A key functional characteristic of PGCs is their slow cell growth and their quiescence nature at the G2 phase of the cell cycle (Sekl et al., 2007; Takehara and Matsui, 2019). We found that DPPA3-mCherry-positive PGCLCs were enriched in the G2 phase of the cell cycle (Figure 4A). One functional property that distinguishes PGCs from ESCs and differentiated cells is their responsiveness to RA, which actively stimulates PGC proliferation while inducing rapid differentiation in ESCs (Dyce et al., 2018; Zhang et al., 2015). We found that the sorted DPPA3-mCherry-positive cells formed alkaline phosphatase-positive colonies in the presence of RA, whereas DPPA3-mCherry-negative differentiated cells did not stain for alkaline phosphatase (Figures 4B,C). Furthermore, we derived continuously growing EGCs by isolating individual RA-resistant colonies that developed from DPPA3-mCherry-positive cells and were grown in LIF-containing ESC growth medium, corroborating previous studies (Leitch et al., 2010; Shamblott et al., 1998). These results further demonstrate the functionality of differentiated PGCLCs in our system.

In summary, we have developed an efficient and simple PGCLC differentiation protocol from mESCs, achieving ∼48% DPPA3-positive PGCLCs compared to ∼10% in previously described methods (Hayashi et al., 2011). This protocol will be useful not only for differentiating functional gametes but also for enabling the characterization of the roles and molecular functions of novel genes in PGC specification *in vitro*.

## Materials and methods

### Generation of the TDM11 cell line and culture

The TDM11 cell line was generated by genetic modification of the E14-TG2A (TG2A) mouse embryonic stem cell line. The TG2A cell line was originally used to generate HPRT-knockout mice (Thompson et al., 1989) and has been maintained under feeder-free conditions on gelatin-coated plates (Smith, 1991). TG2A cells were cultured on 0.1% gelatin-coated tissue culture plates in Glasgow Minimum Essential Medium (GMEM; Gibco, Thermo Fisher Scientific, Waltham, MA, United States) supplemented with 10% ES cell-qualified fetal bovine serum (Gibco, Thermo Fisher Scientific), 0.1 mM nonessential amino acids (Gibco, Thermo Fisher Scientific), 1000 U/mL LIF, 0.1 mM 2-mercaptoethanol (Thermo Fisher Scientific), and 1 mM sodium pyruvate (Sigma-Aldrich, St. Louis, MO, United States). To enable faithful quantification of the live PGCLC, a T2A-mCherry-IRES-Neo cassette was knocked in at the endogenous Dppa3 locus in frame, immediately upstream of the stop codon, using CRISPR–Cas9-mediated gene editing, as depicted in Figure 1A. The targeting plasmid is also depicted in Figure 1A. The two guide RNA oligonucleotides (CAC​CGA​GGT​ATA​ATG​TGT​TGG​CTA​G and CAC​CGT​AAT​TTA​CAC​AAA​CAG​CTA​G), targeting the last exon of *Dppa3*, were cloned under the regulation of the U6 promoter in a separate plasmid also containing SpCas9 [which was developed in previous studies to minimize the off targets (Kleinstiver et al., 2016)], driven by the chicken β-actin promoter.

Targeting the plasmid and dual guide-RNA/SpCas9 vector was co-nucleofected into TG2A cells using a Lonza 4D Nucleofector system (Lonza Bioscience, Cologne, Germany). Following nucleofection, cells were grown in the growth medium overnight and then selected by adding 150 μg/mL neomycin to the growth medium for 7 days. The individual resistant colonies were isolated under a microscope, dissociated using TrypLE (Invitrogen, United States), and propagated. Correctly targeted clones were screened by PCR-based genotyping for site-specific recombination at the *Dppa3* locus, yielding an expected amplificon size of approximately 1.1 kb (Figure 1B).

One of the positive clones, clone number 11, referred to as TDM11 (TG2A Dppa3 mCherry clone 11), was further validated by amplification of the Dppa3-T2A-mCherry fusion transcript from cDNA (Figure 1C). To confirm that the integration was in frame with the Dppa3 gene and that the T2A-mCherry sequence was positioned immediately after the last codon of Dppa3, the Dppa3-mCherry amplicon was sequenced (Supplementary Figure S1A). DPPA3-mCherry fluorescence in the TDM11 clone was verified by flow cytometry using a BD LSRFortessa instrument (BD Biosciences, San Jose, CA, United States) (Figure 1E).

### PGCLC induction from the TDM11 cell line

TDM11 cells were maintained feeder-free under the same culture conditions as parental TG2A cells, as described above. For PGCLC differentiation, approximately 5 × 10^5^ TDM11 cells were seeded into non-adherent P60 culture plates in 3 mL of the differentiation medium (Table 1) to promote EB formation. EB formation was initiated directly from feeder-free ESC cultures. The differentiation medium with varying compositions, including combinations of ascorbic acid, transferrin, BMP4, and BMP8b, was used, as specified in Table 1.

Because EBs are small and susceptible to loss during early medium changes, no medium was removed during the first 2 days of differentiation. Instead, 1 mL of fresh differentiation medium was added daily on days 1 and 2. From day 3 onward, half of the medium (2.5 mL) was replaced daily with fresh differentiation medium until day 8. Growth factors and small-molecule supplementation were applied continuously from day 0 through day 8 of differentiation, unless otherwise indicated.

### Flow cytometric analysis and cell sorting

Cells from adherent cultures and EBs were dissociated using TrypLE enzyme (Thermo Fisher Scientific) and incubated for 5 min at 37 °C in a CO_2_ incubator. The EBs were dissociated into single cells by pipetting several times using a 1-mL pipette tip. The enzymatic activity was quenched by adding 10% FBS. Cells were centrifuged and diluted in PBS for flow cytometric analysis using a BD LSRFortessa flow cytometer (BD Biosciences, San Jose, CA, United States). TG2A cells were used as a negative control for mCherry fluorescence. The data were analyzed using FlowJo software (BD Biosciences, Ashland, OR, United States).

For sorting mCherry-positive and -negative cells, the dissociated cells were sorted in basal medium using a BD FACSAria Fusion flow cytometer (BD Biosciences, San Jose, CA, United States). The sorted cells were used for further analysis or characterization.

### Quantitative PCR

Gene expression analysis was performed using quantitative PCR (qPCR). RNA from samples was isolated manually using TRIzol reagent (Thermo Fisher Scientific) and diluted in nuclease-free water. Approximately 1,000 ng of RNA was converted into cDNA using a cDNA synthesis kit, following the manufacturer’s instructions (Takara Bio Inc., Shiga, Japan). qPCR was performed using Power SYBR Green Master Mix (Thermo Fisher Scientific) and gene-specific primers. The PCR reaction was carried out on an ABI ViiA™ 7 Real-Time PCR System (Thermo Fisher Scientific), as described previously (Kumar et al., 2024).

Ct values for target genes were normalized by subtracting Ct values of the GAPDH gene in each sample. Fold change was calculated using the formula 2^^−^(normalized Ct value of the test sample − normalized Ct value of the control sample). The fold change was plotted from three biological replicates.

### Immunoblotting

Cell lysates were prepared from cells or EBs using RIPA lysis buffer supplemented with cOmplete protease inhibitors (Roche Diagnostics, Basel, Switzerland) and incubated on ice for 30 min. The lysates were further sonicated for 5 min using 30-s on/off cycles. Remaining cell debris was removed through centrifugation. Protein concentration was estimated, and proteins were separated based on size using gradient polyacrylamide gel electrophoresis.

Proteins were transferred onto PVDF membranes and probed using the indicated antibodies. The blots were developed using enhanced chemiluminescence (SuperSignal, Thermo Fisher Scientific) and imaged under a ChemiDoc Imaging System (Bio-Rad Laboratories, Hercules, CA, United States).

### Derivation of EGCs from PGCLCs

After growing EBs in differentiation medium for 5 days, the cells were dissociated into a single-cell suspension, and DPPA3-mCherry-positive and -negative cells were sorted using FACS. Approximately 3,000 cells per well were seeded on mitotically inactivated mouse embryonic fibroblasts (MEFs) in a 6-well plate and cultured in the previously described EGC medium (GMEM, 10% FBS, 1 mM sodium pyruvate, 0.1 mM nonessential amino acid, 1 mM GlutaMax, 0.1 mM 2-mercaptoethanol, 2 µM retinoic acid, 1000 units/mL LIF, 30 ng/mL SCF, and 15 ng/mL bFGF) (Vincent et al., 2011). The medium was replenished every day, and after 5 days of growth, the grown colonies were either harvested or assayed for alkaline phosphatase activity. The harvested cells were further propagated under ESC culture conditions.

### Cell cycle analysis

The cells were sorted from differentiated EBs on day 5. The sorted cells from differentiating EBs were fixed using 70% ice-cold ethanol and stained with propidium iodide. The stained cells were treated with RNase to remove RNA for 15 min and analyzed using a flow cytometer. The cell cycle was plotted using FlowJo software.

## Data Availability

The original contributions presented in the study are included in the article/Supplementary Material; further inquiries can be directed to the corresponding authors.
