# Supplementary material for: Ascorbic acid enhances in vitro primordial germ cell-like cell differentiation from mouse ESCs
Source: Front Cell Dev Biol. 2026 Mar 4;14:1755998. doi: 10.3389/fcell.2026.1755998 (PMC12996197; doi:10.3389/fcell.2026.1755998)

**Supplementary figure S1- Sanger sequencing validation of *Dppa3*-T2A-mCherry transcript**.
Strategy for amplification of the *Dppa3*-T2A-mCherry transcript from cDNA of clone 11 (TDM11) using a forward primer located in the first exon of *Dppa3* (untargeted sequence) and a reverse primer located within the mCherry sequence. The chromatogram shows the sequenced *Dppa3*-T2A-mCherry transcript confirming correct in-frame fusion.


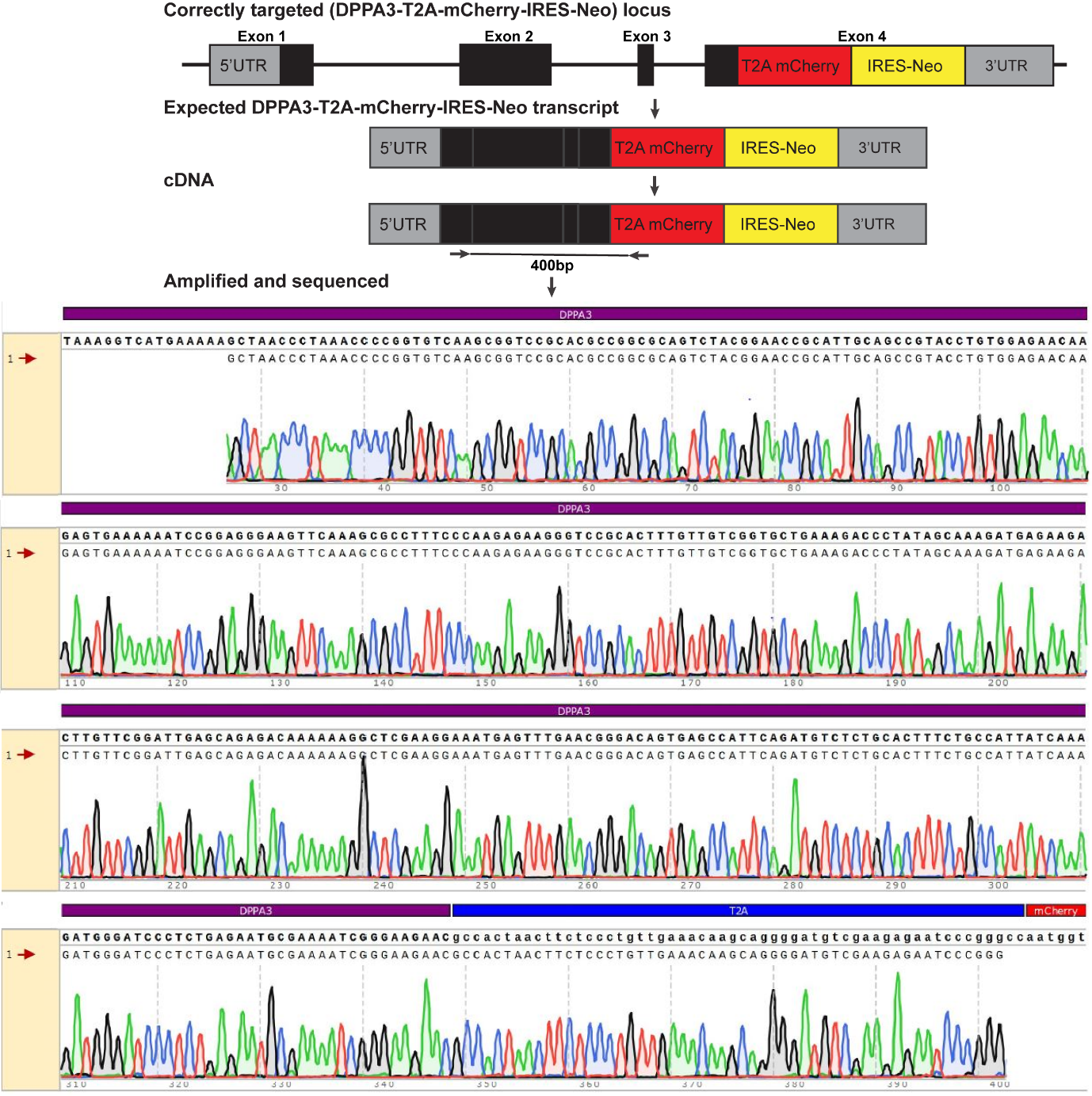


**Supplementary figure S2: Flow cytometric analysis of *Dppa3*-mCherry–positive cells during differentiation.**(A) Flow cytometric analysis of TDM11 cells differentiated in Basal medium, Basal medium supplemented with ascorbic acid (AA), or complete differentiation medium for up to 8 days. (B) The percentage of *Dppa3*-mCherry–positive cells was quantified and plotted for each condition.

**A**


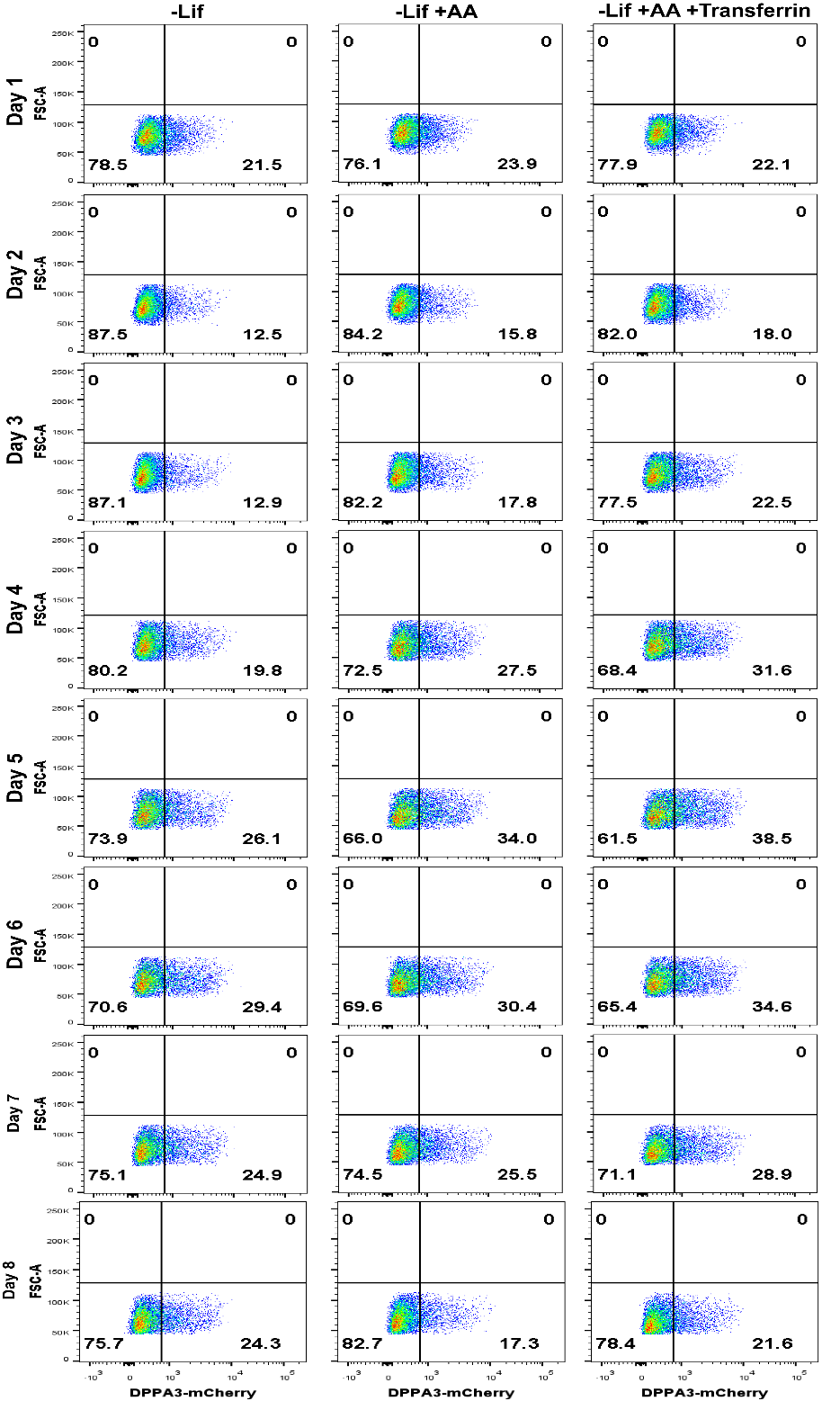


Basal medium

Basal medium + AA

Differentiation medium

**B**


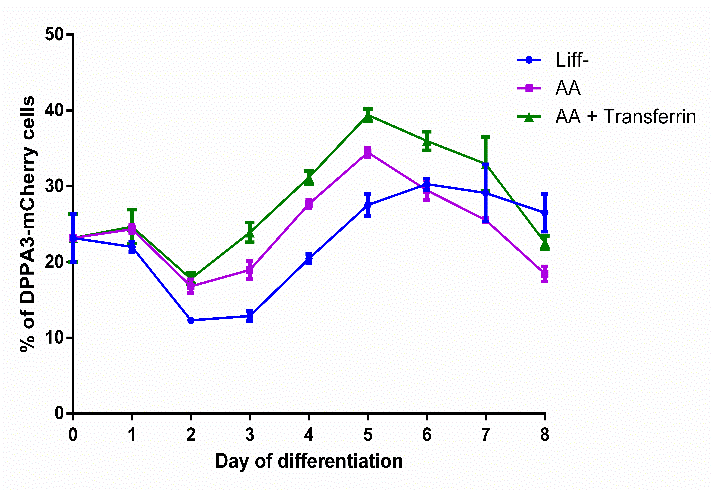


**Basal medium + AA**

**Basal medium**

**Differentiation medium**

**Supplementary figure S3: Uncropped blots used in figure 1G.**

Original uncropped western blots of NANOG, OCT4, SOX2, DPPA3 and β Actin used in figure 1G


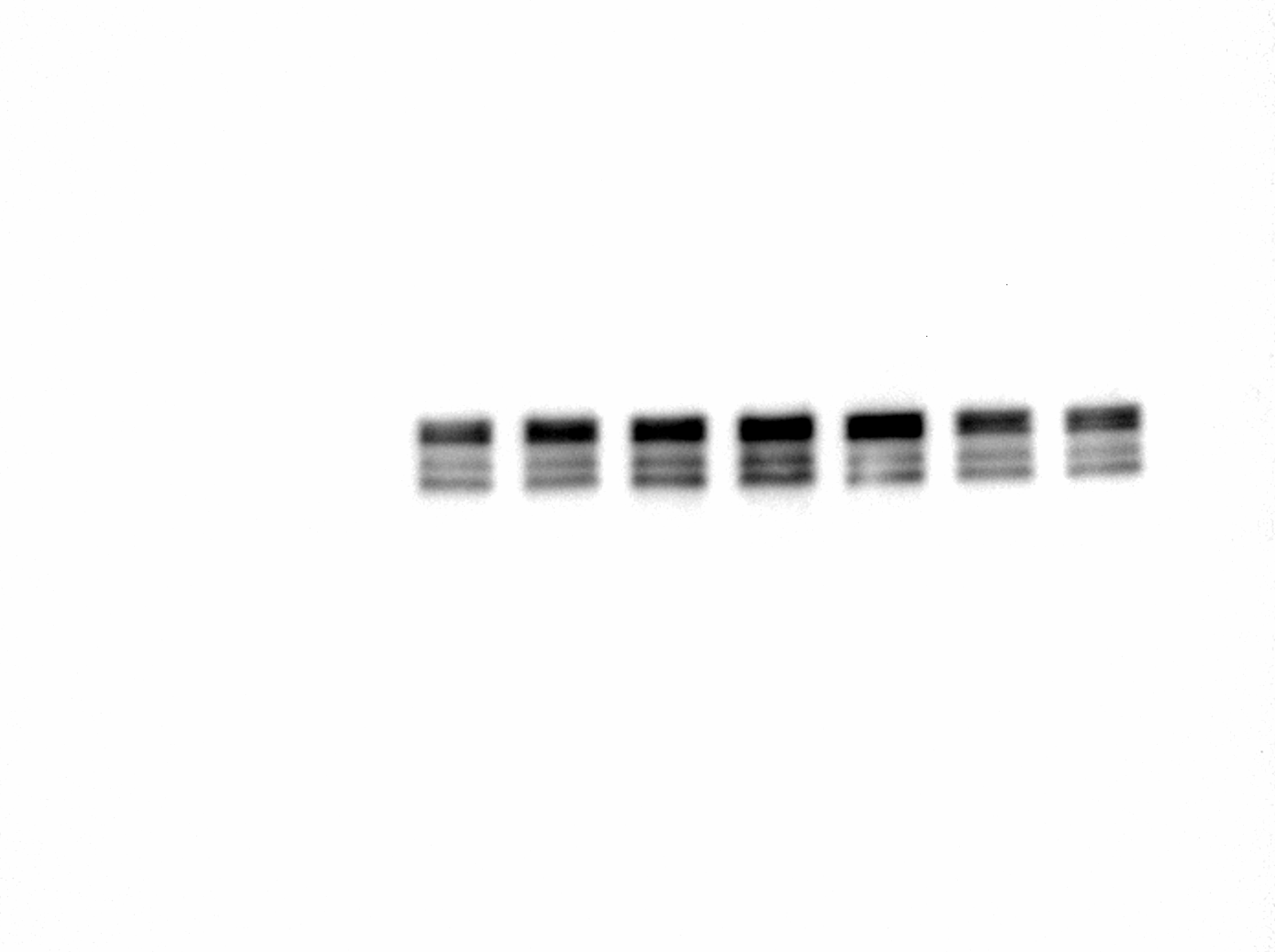


NANOG


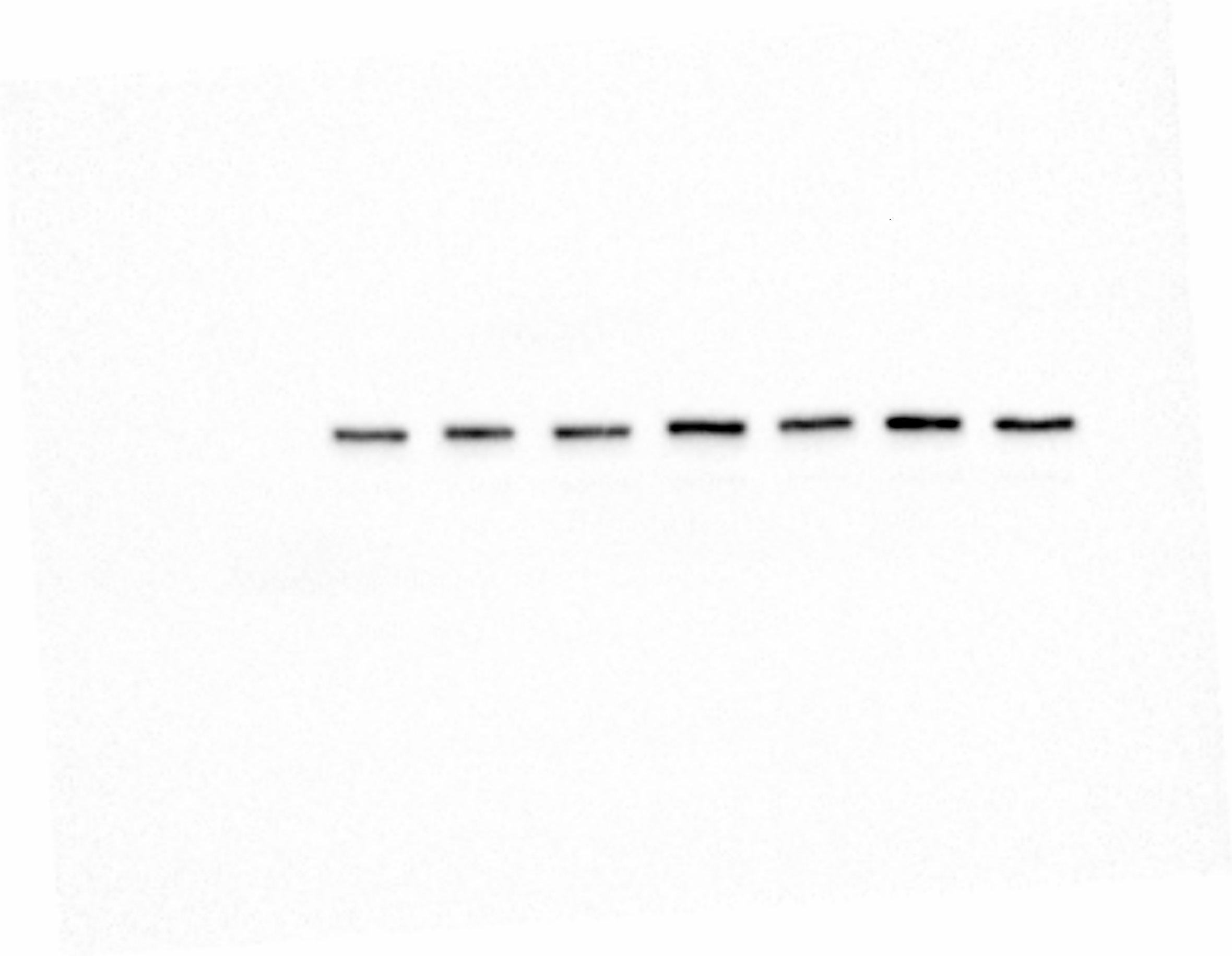


OCT4


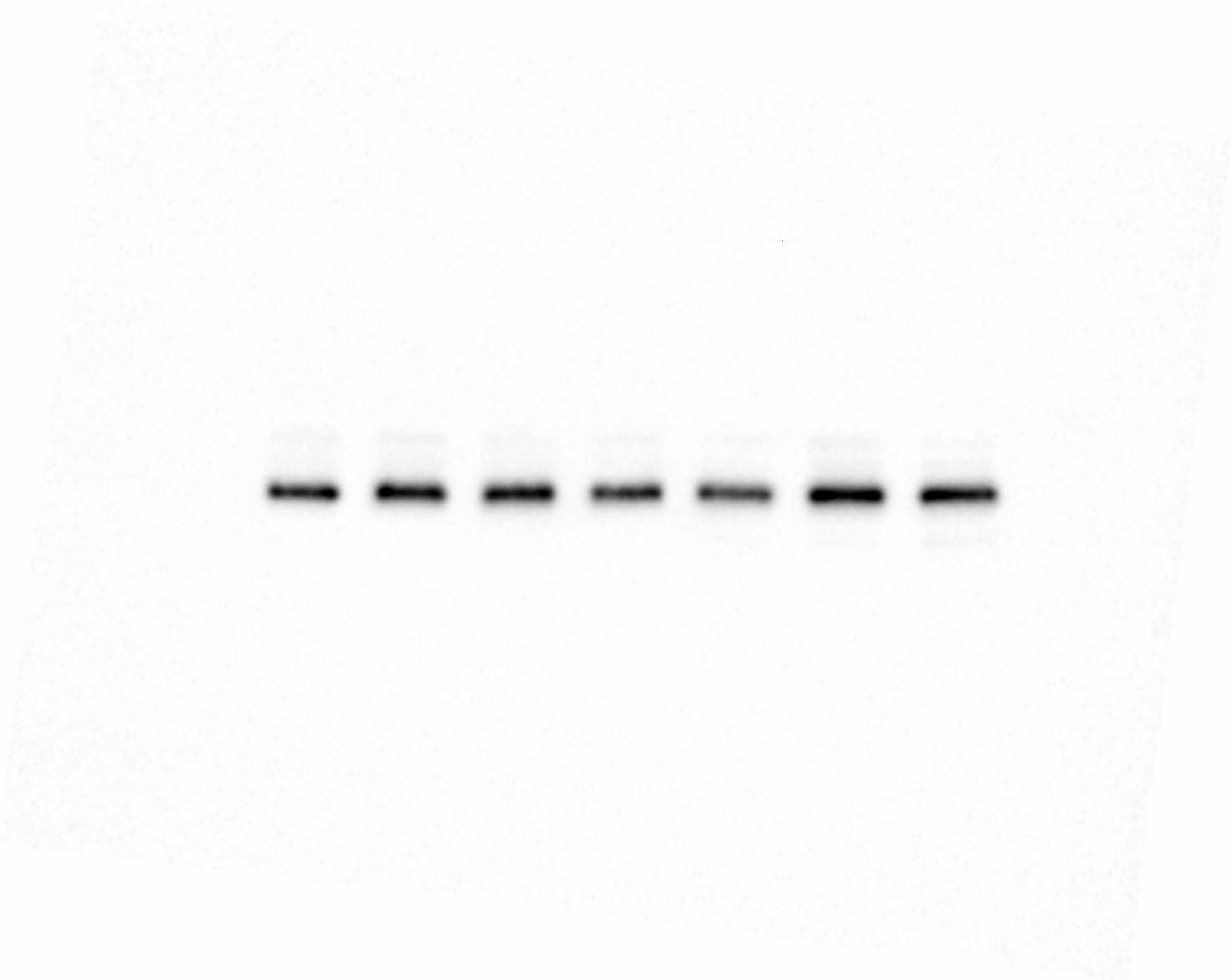


SOX2


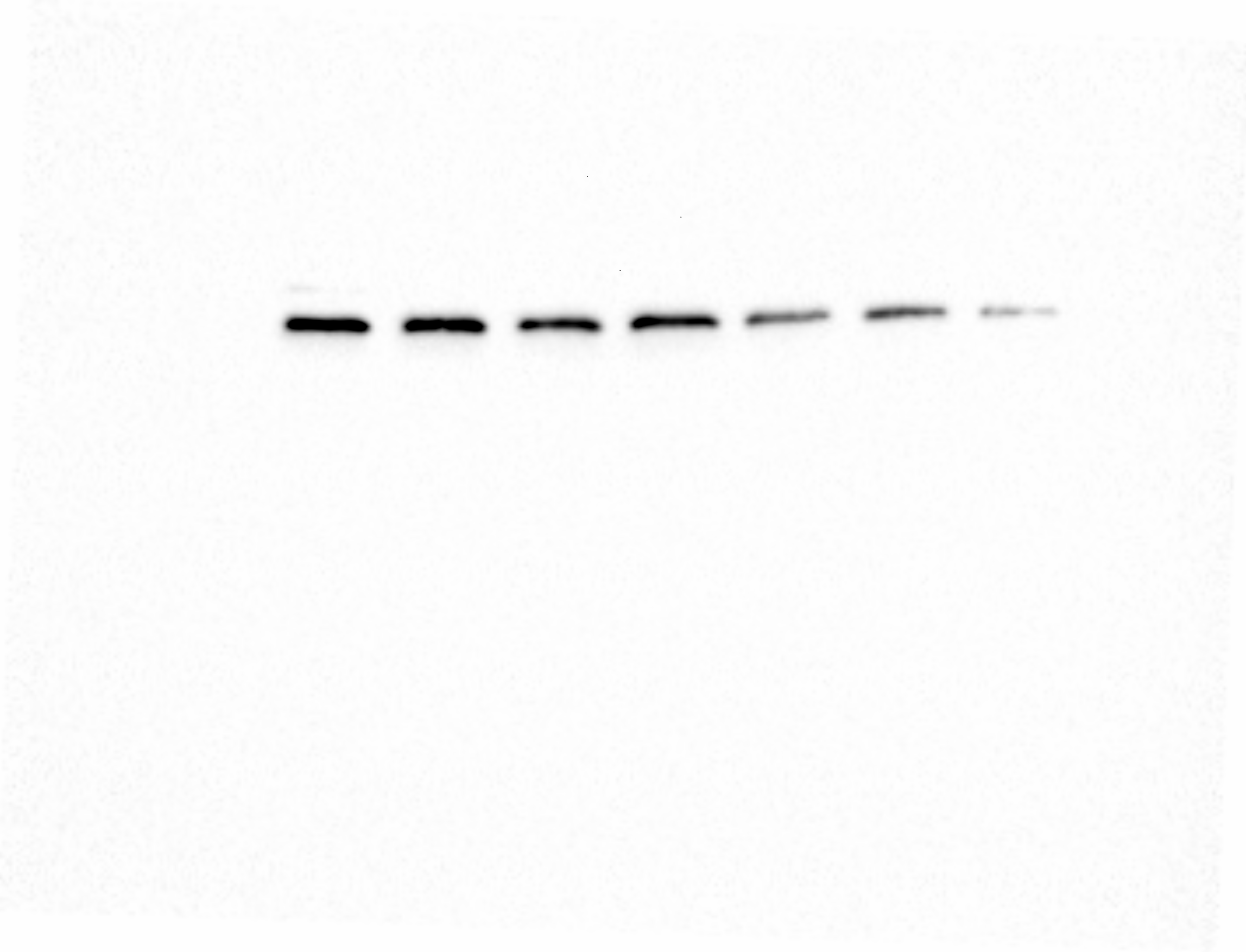


DPPA3


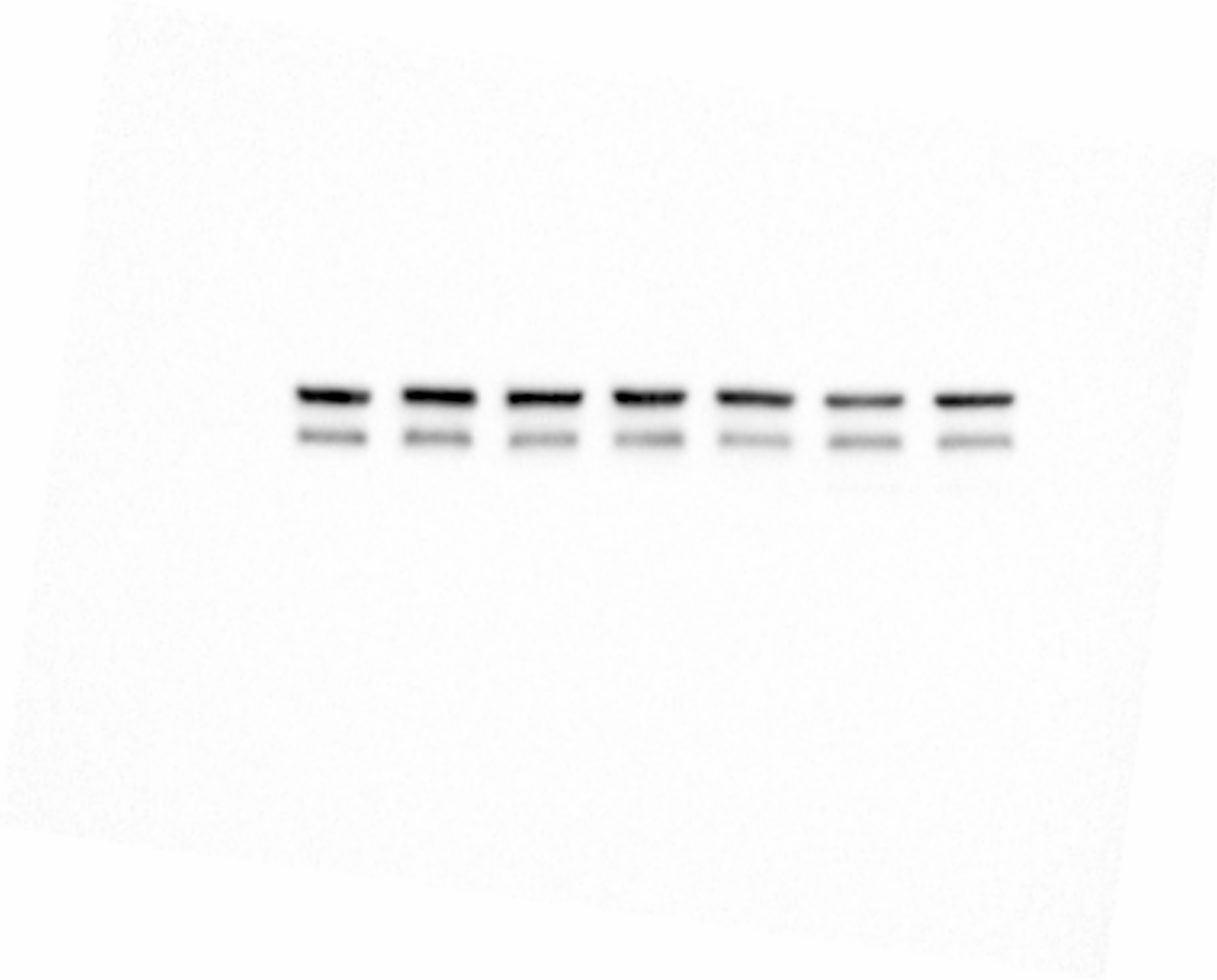


β Actin

TG2A

TDM11

TG2A

TDM11

TG2A

TDM11

TG2A

TDM11

TG2A

TDM11

**Supplementary figure S4: Uncropped blots used in figure 2E**

Original uncropped western blots of DNMT3B, INTEGRIN β3, OCT4, SOX2, DPPA3, NANOS2 and GAPDH used in figure 2E


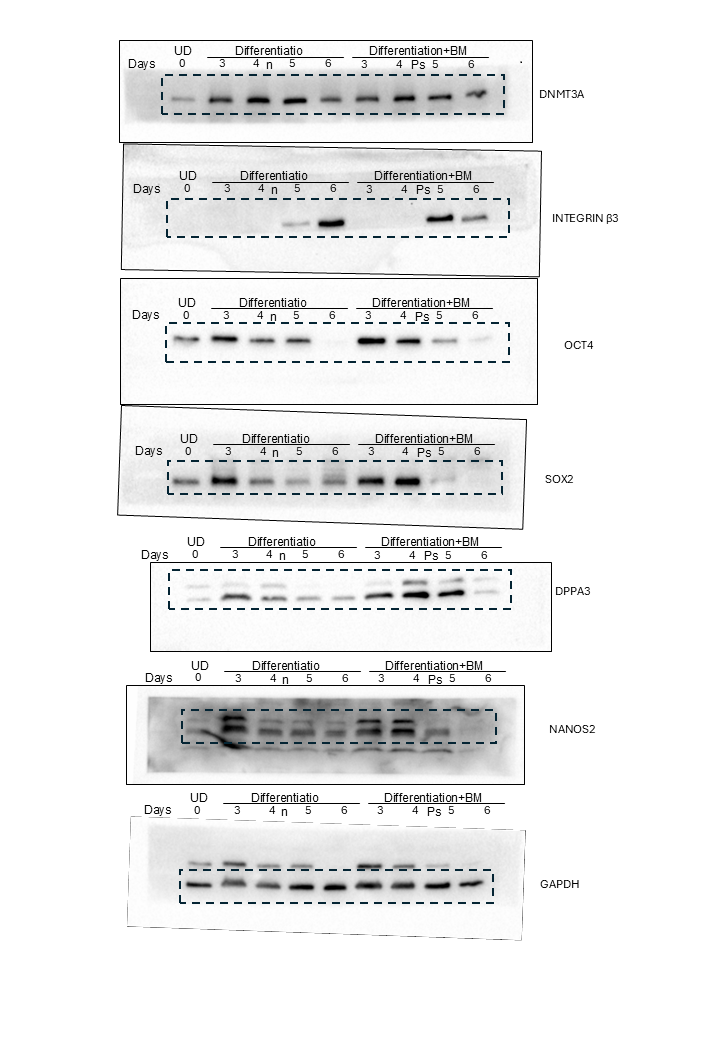

Supplement: Supplementary file 1 [file DataSheet1.docx]
